# A six years’ experience with 41 cases of enterovesical fistula in a Tertiary National Hospital in Indonesia: A retrospective study

**DOI:** 10.1016/j.amsu.2021.103102

**Published:** 2021-11-23

**Authors:** Fina Widia, Muhammad Firman, Gampo Alam Irdam, Ridho Ardhi Syaiful

**Affiliations:** a Department of Urology, Faculty of Medicine Universitas Indonesia, Jl. Diponegoro No. 71, Salemba, Jakarta Pusat, DKI Jakarta, 10430, Jakarta, Indonesia; b Department of Surgery, Faculty of Medicine Universitas Indonesia, Jl. Diponegoro No. 71, Salemba, Jakarta Pusat, DKI Jakarta, 10430, Jakarta, Indonesia

**Keywords:** Intestinal fistula, Urinary fistula, Enterovesical fistula, Surgery

## Abstract

**Introduction:**

The incidence of Enterovesical Fistula (EVF) is relatively low. Currently, there is no agreement about the best methods for EVF management. This study was performed to investigate the characteristics of EVF to find the optimal diagnostic and management pattern.

**Methods:**

Data were collected retrospectively from the medical record at Cipto Mangunkusumo Hospital. Patients diagnosed with EVF between January 2014 and April 2019 were included. They were evaluated for demographics, characteristics, diagnostic modalities, and treatment modalities.

**Results:**

From 41 patients, 26 (63.3%) are male, and 15 (36.6%) are female. Peak incidence was 51–60 years old. The most common symptoms are fecaluria found in 32 (78%) patients. The common etiology is gastrointestinal cancer found in 17 (41.5%) patients, followed by gynecologic cancer and diverticulitis found in both 9 (22%) patients. The rectovesical fistula was seen in 25 (61%) patients with an advanced stage rectosigmoid cancer, followed by colovesical in 14 (34.1%) of patients with sigmoid diverticulitis (p 0.038). The common diagnostic modalities performed are cystoscopy in 32 (78%), followed by colonoscopy in 11 (26.8%) patients. The preferred modalities that were used in most cases were surgery in 35 (85.4%) patients. A two-stage surgical approach was used in 28 (68.3%) patients.

**Conclusion:**

The incidence of EVF is uncommon. Malignancy was the leading cause of EVF in this study. Combined diagnostic modalities are recommended in EVF cases. The two-stage surgical approach was the preferred modality. Further prospective studies are mandatory to analyze this condition.

## Highlights

• - Enterovesical fistula (EVF) incidence is relatively low with no uniformly agreed management.
• - Malignancy was the most common cause of the fistula.
• - The underlying disease were significantly correlate to the anatomical location of the fistula (p-value = 0.038).
• - Surgical approach (Two-stage) was the preferred modality used in most cases.

## 1 Introduction

Enterovesical fistula (EVF) is an anatomical disorder where there is a connection between the bladder and the intestine [1,2]. It is frequently seen in bowel disease, which strongly influences the type of fistula [1,3]. Based on the part of the bowel involved, EVF is divided into four categories, namely colovesical, rectovesical, ileovesical, and appendicovesical EVFs [3].

EVF is estimated to occur in one person for every 3.000 surgical admissions. In 60–70% of cases, EVF is caused by diverticulitis strongly associated with the colovesical fistula [1,2]. The second most common etiology is advanced stage colorectal cancer (10–20%), which is mainly located in the rectosigmoid but also occurs as a complication of other malignancies in the pelvic organ [4,5]. Less common etiology is Crohn's disease (5–7%), with ileovesical fistula as the most common type of fistula [1,5]. The other causes are trauma, iatrogenic, appendiceal abscess, and foreign body [3,5].

The diagnosis of EVF poses a significant challenge. In most cases, patients are observed for months before the condition is known and treated effectively. Therefore, this condition causes substantial morbidity and decreases the quality of life in the patients.

Because of its relatively low incidence, there are no current approved guidelines for optimal methods of management [6]. In this study, we described the characteristic of enterovesical patients and the approaches used to find the optimal diagnosis and management of this disease.

## 2 Methods

The data of this case series study were collected retrospectively from the medical record at Cipto Mangunkusumo Hospital. The inclusion criteria are all the patients that were diagnosed with EVF between January 2014 and April 2019. Medical records of these patients were evaluated for demographics, symptoms, underlying cause, fistula site, diagnostic modalities, treatment modalities, and the surgical approach. The exclusion criteria are uncompleted medical data.

The data were presented descriptively. Categorical data were presented as an absolute value and percentage. Numerical data were presented as mean and standard deviation if the data had normal distribution or median and range if the data did not have a normal distribution. All the data were processed using Statistical Package for the Social Science (SPSS) version 23. This case series has been reported in line with the PROCESS Guideline [7], and is registered with the Research Registry, and the unique identifying number is: researchregistry7166 [8].

## 3 Results

A total of 41 patients were included in this study. 26 (63.3%) patients are male, and 15 (36,6%) patients are female, with a male to female ratio of 2:1. The mean age was 53.51 (±15.74) years. In male and female patients, the EVF cases are not common at a young age; instead, their incidence rises through the period. The peak incidence of EVF occurred at 41 - 60 years old, as shown in Table 1.

**Table 1:** EVF distribution according to age and gender.

	Number of Patients, *n*	Percentage of patients, *%*
Gender		
*Male*	26	63.3
*Female*	15	36.6
**Age (years)**		
*≤20*	2	4.8
*21 – 40*	3	7.3
*41 – 60*	23	56.0
*>60*	13	31.7
**Underlying disease**		
*Gastrointestinal cancer*	17	41.5
*Diverticulitis*	9	22
*Gynecologic cancer*	9	22
*Crohn's disease*	1	2.5
*Others*	5	12.2
**Symptoms**		
*Faecaluria*	32	66,6
*Urinary tract infection (UTI)*	6	12,5
*Bladder pain*	3	6,3
*Urinary incontinence through the vaginal (females only)*	3	6,3
*Others*	4	8,3

The most common emerging symptoms found in 32 (66,6%) patients is fecaluria, followed by urinary tract infection (UTI) in 6 (12,5%) patients and bladder pain in 3 (6,3%) patients. In females, 3 (6.3%) patients were seen having urinary incontinence through the vaginal.

The underlying cause of the EVF was divided into five categories. Gastrointestinal cancer was seen as the most common cause of EVF in 17 (41.5%) patients, including the advanced stage of rectosigmoid adenocarcinoma. It is followed by gynecologic cancer found in 9 (22%) patients, including cervical carcinoma IIIB, endometrial carcinoma, and ovarian carcinoma. Other common causes include diverticulitis of sigmoid with a Hinchey III classification found in 9 (22%) patients, Crohn's disease found in 1 (2.5 %) patient, and others, including complication from previous surgery, anorectal malformation, and chronic infection, found in 5 (12.2%) patients.

The fistula's most common type and anatomical location in the patients are rectovesical found in 25 (61%) patients. It is followed by colovesical found in 14 (34.1%) patients and ileovesical found in 2 (4.8%) patients. Furthermore, 5 (20%) patients with rectovesical fistula were seen having a vaginal involvement. In colovesical fistula patients, 2 (14.2%) patients were also seen involving the vagina, and 1 (7.1%) patient was seen having cutaneous involvement [Table 2].

**Table 2:** Anatomical location of the fistula.

	Total		Male		Female	
	n	%	n	%	n	%
**Rectovesical**	25	61	15	60	10	40
**- Rectovesicovaginal**	5	20	0	0.	5	50
**Colovesical**	14	34.1	9	64.3	5	35.7
**- Colovesicovaginal**	2	14.2	0	0.	2	40
**- Colovesicocutaneus**	1	7.1	0	0.	1	20
**Ileovesical**	2	4.8	2	7.6	0	0.

Based on the underlying cause of the EVF, the anatomical location of the fistula was analyzed as follows. In rectovesical fistula, 11 (55.5%) patients were seen in an advanced stage of rectosigmoid cancer, 4 (20%) patients were seen in diverticulitis sigmoid, 3 (15%) patients were seen in endometrial cancer (2 patients), and ovarian cancer (1 patient), and 2 (10%) patients were seen in others. In colovesical fistula, 5 (45.5%) patients were seen in sigmoid diverticulitis, 3 (27.3%) patients were seen in an advanced stage of rectosigmoid cancer, 1 (9.1%) was seen in Crohn's disease, and 2 (18.2%) patients were seen in others. Ileovesical fistula was seen in 2 patients with advanced stage of rectosigmoid cancer. In rectovesicovaginal fistula, 4 (80%) patients were seen in cervical cancer (3 patients) and ovarian cancer (1 patient), and 1 (20%) patient was seen in others. The colovesicovaginal fistula was seen in 2 patients with cervical cancer. Moreover, the colovesicocutaneous fistula was seen in 1 patient with an advanced stage of rectosigmoid cancer. A significant *p-value* was noted in this category (0.038), as shown in Table 3.

**Table 3:** Anatomical location of fistula based on etiology.

	Diverticulitis		Advanced Stage Cancer				Crohn's disease		Others	
			Gastrointestinal		Gynecologic					
	n	%	n	%	n	%	n	%	n	%
**Rectovesical**	4	20	11	55.5	3	15	0	0.	2	10
**Colovesical**	5	45.5	3	27.3	0	0.	1	9.1	2	18.2
**Ileovesical**	0	0.	2	100	0	0.	0	0.	0	0.
**Rectovesicovaginal**	0	0.	0	0.	4	80	0	0.	1	20
**Colovesicovaginal**	0	0.	0	0.	2	100	0	0.	0	0.
**Colovesicocutaneus**	0	0.	1	100	0	0.	0	0.	0	0.
***p-value***	0.038									

^a^*p-value* was analyzed with chi-square; *p-value* <0,05 means significant.

The first and most common diagnostic modality that was used to diagnose EVF is endoscopy. Cystoscopy was performed on 32 (78%) patients, while colonoscopy was performed on 11 (26.8%) patients. In the patients having cystoscopy, a biopsy was also performed in 29 (90.2%) patients. Biopsy was often performed in a patient with the suspected cause of the fistula from malignancy. The other modalities are imaging study, CT-Scan was completed in 9 (22%) patients, cystography in 9 (22%) patients, and fistulography in 2 (4.9%) patients. The preferred modality used in most cases is surgery performed on 35 (85.4%) patients for the treatment. The two-stage surgical approach used was performed on 28 (68.3%) patients, and a single-stage surgical procedure was performed on 7 (17.1%) patients. One of the patients with a single-stage surgical approach was performed with laparoscopic but was then converted to open surgery. The non-surgery modalities were used in 3 (7.3%) patients, while 3 (7.3%) patients were waiting for definitive treatment.

## 4 Discussion

The incidence of EVF is uncommon. In our study, a total of 41 patients with EVF were identified. The mean age is 53.51 (±15.74) years, with a peak incidence is 51–60 years old in men and >60 years old in women. Similar results by Yehonatan et al. [6] show that the mean age of EVF is 48 (20–75) years old, while Scozzari et al. [3] show that the peak incidence of EVF occurs at 6th and 7th decades of life. Concurrent comorbidities and nutritional status are believed to be a risk factors in the elderly.

In this study, the male to female ratio is 2:1. Shuali Li et al. [2] reported that the male to female ratio is 3:1. These indicate that females are less likely to have an EVF because the uterus divides the bladder and colon's anatomical interposition. However, in some conditions, such as a patient with a history of hysterectomy, there is a significantly increased risk for women to have EVF [9].

The symptoms of EVF may originate from both the urinary and the GI tract. Many studies show different results. Badjani et al. [1] showed the most common sign noted in 50–70% of cases is pneumaturia. Tomasz et al. five also reported pneumaturia in 50–70% of the patients as the common symptoms. Meanwhile, Hsieh JH et al. [10] reported recurrent urinary tract infection in 73% of the patients as the most frequent symptom. Another study by Liu CH et al. [11] reported a different result showing that the most frequent symptom was fecaluria in 58.5% of the cases. In this study, fecaluria was the most common presenting symptom in 78% of patients. Demographic variety in patients that were taken into the study may contribute to these findings.

In this study, the advanced stage of rectosigmoid cancer was the most common underlying disease of EVF in 41.5% of the patients. On the contrary, Scozari et al. [3] reported that diverticulitis was the most common etiology in 50–70% of patients. Another study from Badjani et al. also mentioned that diverticular disease, especially inflammatory diverticula, was the most common finding in 70% of cases. In this study, diverticulitis sigmoid was found in only 22% of patients. These findings mainly because Jakarta have the highest incidence of colorectal cancer in Indonesia [12], and diverticular disease is uncommon in developing nations [13].

The most common anatomical location seen in this study is rectovesical fistula in 61% of patients, 60% of cases in males, and 40% in females. Fistulas that involved three anatomical locations were also common findings in the study. In females, rectovesicovaginal was the most common finding in 50% of patients.

The anatomical location found based on the underlying disease in this study shows a significant result with a *p-value* = 0.038. This is in line with Badjani et al. stating that 65–79% of the diverticular cases were strongly correlated with colovesical fistula. In this study, the colovesical fistula was the common finding in 45.5% of patients with diverticulosis. In rectovesical fistulas, the underlying cause is almost always due to malignancy or trauma [7]. This study shows similar results in which 55.5% of patients with rectovesical fistula were caused by gastrointestinal cancer. In addition, ileovesical, colovesicocutaneus, and gastrointestinal cancers were the underlying causes. While in females, rectovesicovaginal, colovesicovaginal fistula and gynecologic cancers were the most common findings in 80% of patients and 100% of patients.

A lot of modalities are available for diagnosing EVF. In this study, the modality commonly used for assessing patients is cystoscopy found in 78% of the cases. Biopsy for fistula margin during cystoscopy is also possible which is useful since most of the underlying disease for fistula in our center is cancer. The surgeon can also see the location of the fistula and its relation to ureter orifice while doing cystoscopy. Badjani et al. [1] reported that cystoscopy had the highest yield in identifying the potential lesion but was often nonspecific for making a definitive diagnosis. Therefore, the primary imaging modality used for EVF is CT-Scan due to its high sensitivity and specificity for detection. It is also less invasive and may provide essential findings that help the diagnosis, such as bladder wall thickening adjacent to a loop of thickening colon, presence of colonic diverticula, and the critical finding of intravesical air [1,5,8]. However, in this study, a CT scan was performed only in 22% of the patients. Shuai Li et al. recommend performing CT scan with other modalities, especially endoscopic cystoscopy, colonoscopy, cystography, and barium enema, to increase the detection rate of EVF [2].

The treatment for EVF was divided into two approaches, non-operative and operative. The non-operative approach is safe and preferred as the initial approach in the selected patient, which unfits for primary intervention or in an extensive malignancy process [3,5]. It mainly focuses on parenteral nutrition, bowel rest, and an antibiotic if needed or without active interventions for years [1,3,5]. In this study, 7.3% of the patients were managed conservatively. Radwan R et al. reported no difference in mortality in 26 patients treated conservatively in a study [14].

In this study, the most preferred modality used is the operative approach performed to 85% of the patients. Badjani et al. divided the operative system for EVF into one, two, or three-stage procedures. The one-stage procedure involves fistula removal, closure of involved organs, resection of fistula part in the intestine (Hartmann procedure), and primary re-anastomosis of the resected bowel. In a two-stage process, the first step involves the removal of the fistula, resection of involved organs, and creating diverting colostomy or end colostomy. The second step consists of the takedown of the colostomy once the fistula is healed. In a three-stage procedure, especially for complex fistula involving more than two organs or sites, the first thing to do is diverge both the GI tract and urinary tract. It is followed by the recovery stage, including parenteral nutrition, organ support, and surgical planning. Finally, it was a multidisciplinary joint urologic and digestive surgeon in reconstructive surgery [1,3,5]. The preferred surgical approach used in this study is the two-stage surgical approach, performed in 68.3% of the cases. The most common underlying disease for EVF is tumor, which making it difficult to ensure the surgical incision margin to be clear from the remaining tumor. The other reason is in our center, patients often came with decrease Karnofsky score and decline in health and well-being, thus it is not suitable to choose single-stage approach. In intraoperation setting, oedema tissue and inflammation is frequently found, thus making the two-stage approach becomes a better choice. This approach was chosen based on the characteristics of the fistula, the surrounding bladder tissue, and surgeon preference. [1,3,6].

## 5 Conclusion

The incidence of EVF is uncommon. Malignancy was the leading cause of EVF in this study. Combined diagnostic modalities are recommended in the EVF case. The two-stage surgical approach was the preferred modality in this study. The methods used were highly influenced by surgeon preference. Further prospective studies are mandatory to analyze this condition.

## Provenance and peer review

Not commissioned, externally peer-reviewed.

## Ethical approval

Ethics for this study was not available.

## Sources of funding

This research received funding from PUTI Universitas Indonesia.

## Author contribution

**Fina Widia** = writing paper**,** data analysis, data collection, study concept.

**Muhammad Firman** = data collection, writing paper, study concept, data analysis.

**Gampo Alam Irdam** = writing paper, data analysis.

**Ridho Ardhi Syaiful** = writing paper, data analysis.

## Consent

Consents from patients in this study has not been obtained because this is secondary study.

## Registration of Research Studies

1. Name of the registry: research registry.

2. Unique Identifying number or registration ID: researchregistry7166.

3. Hyperlink to your specific registration (must be publicly accessible and will be checked): www.researchregistry.com/register-now#home/registrationdetails/6147478044fce8001e1c84fc/

## Guarantor

Fina Widia, MD, Finawidia.urology@gmail.com.

## Declaration of competing interest

There is no conflict of interest, neither financial nor nonfinancial, in the whole process from making to publishing this study.
